# ﻿*Drepanogynis insciata* (Felder & Rogenhofer, 1875), a South African geometrid moth lost to science rediscovered after more than 140 years (Lepidoptera, Geometridae, Ennominae)

**DOI:** 10.3897/zookeys.1261.171904

**Published:** 2025-12-02

**Authors:** Pasi Sihvonen, Kyung Min Lee, Max Söderholm, Hossein Rajaei, Axel Hausmann, Hermann S. Staude

**Affiliations:** 1 Finnish Museum of Natural History, P. O. Box 17 (Pohjoinen Rautatiekatu 13), FI-00014, University of Helsinki, Helsinki, Finland University of Helsinki Helsinki Finland; 2 State Museum of Natural History, Stuttgart, Germany State Museum of Natural History Stuttgart Germany; 3 Bavarian State Collection of Zoology (SNSB-ZSM), Munich, Germany Bavarian State Collection of Zoology (SNSB-ZSM) Munich Germany; 4 Duif str. 2, Hoekwil, South Africa Unaffiliated Hoekwil South Africa

**Keywords:** DNA barcoding, molecular phylogeny, morphology, rediscovery

## Abstract

The geometrid moth *Drepanogynis insciata* (Felder & Rogenhofer, 1875) has been known only from two specimens in the Natural History Museum, London, UK: the holotype male collected before 1875 from South Africa, and a second male specimen without date and collecting locality data but probably collected before 1879. Unexpectedly, 13 specimens of this species—long considered lost to science—were observed in four locations between 2020 and 2023 in the Western Cape, near the type locality of the species. These observations were based on photographs available on iNaturalist, apart from a single male attracted to light, which was collected and vouchered for study from the Western Cape on 28 January 2022. We illustrate *D. insciata* and the newly collected specimen using both classical dissection and non-destructive micro-CT imaging. Phylogenetic analysis based on the multi-gene maximum-likelihood method places the species within Ennominae, tribe Drepanogynini. DNA barcodes reveal its nearest genetic neighbor to be *Drepanogynis smaragdaria* Krüger, 2002, with 5.9% sequence divergence. We discuss the conservation implications of this rediscovery.

## ﻿Introduction

Occasionally, rare or presumed extinct species are rediscovered, and such cases often make headlines. Recent examples include the Portuguese spider (*Nemesia berlandii* Frade & Bacelar, 1931; Arachnida, Nemesiidae), not discovered since 1931 and found again in 2023; the earless Australian dragon (*Tympanocryptis pinguicolla* Mitchell, 1948; Sauropsida, Agamidae), last seen in 1969 and rediscovered in 2023; and Wallace’s giant bee from Indonesia (*Megachile pluto* (Smith, 1860); Hymenoptera, Megachilidae), which had vanished since 1981 and was rediscovered in 2019 (Rewild 2025). The Critically Endangered South African geometrid moth *Callioratis millari* (Hampson, 1905) disappeared when its known habitat was destroyed in 1928 and was not found, despite extensive searching, until 1996 when it was rediscovered over 100 km north of its type locality (Staude 2001).

**Figure 1.**
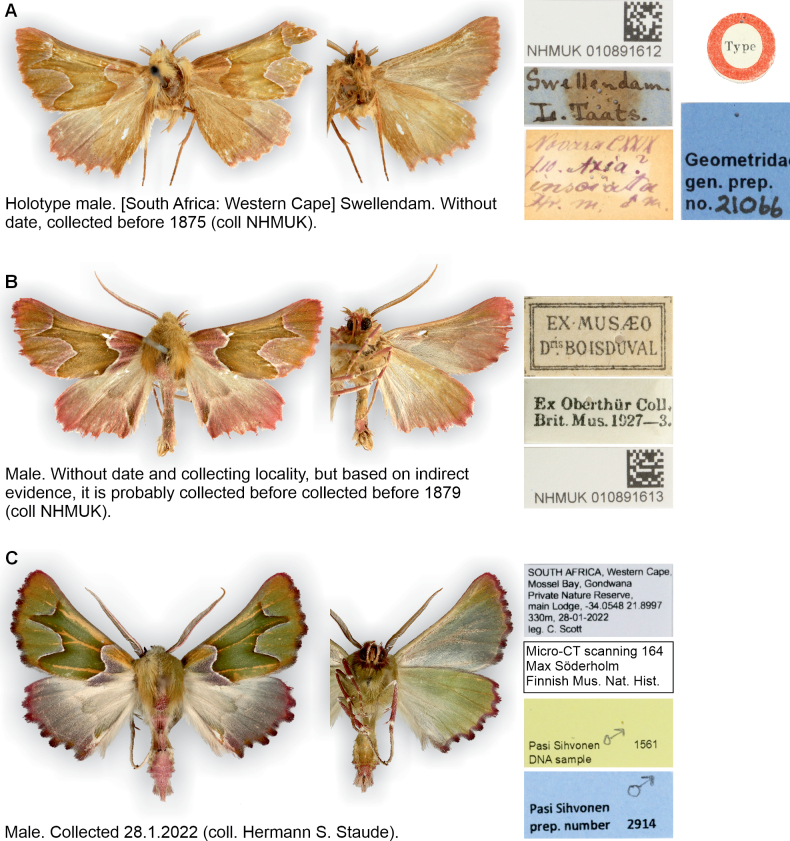
External characters and selected labels of all known *Drepanogynis insciata* specimens. **A.** Holotype male, without date, but specimen collected before 1875; **B.** An old specimen in The Natural History Museum, London, UK. The specimen is without date and location data, but based on indirect evidence, it is probably collected before 1879; **C.** Specimen collected on 28 January 2022 from Western Cape: Gondwana Private Game Reserve, after 147 years when the species was described. The old specimens have lost their green color.

**Figure 2.**
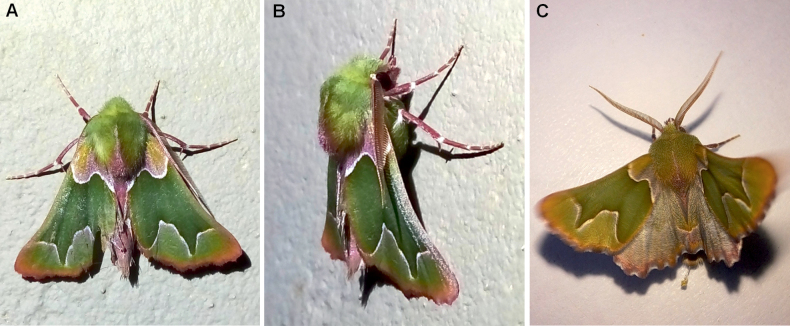
A selection of *Drepanogynis insciata* male specimens available on iNaturalist. The physical specimens were not vouchered for study, but the identity of these, judged by the external characters, agrees with typical *D. insciata*. All specimens are from South Africa: Western Cape district. **A.** Mossel Bay district, Hartebeestkuil Game Reserve, 10 November 2023, dorsal view (photo by Eon Grobler); **B.** Mossel Bay district, Hartebeestkuil Game Reserve, 10 November 2023, lateral view (photo by Eon Grobler); **C.** Mossel Bay, Gondwana Private Game Reserve, 7 September 2023 (photo by Jolandie3). Photos are reproduced under CC BY-NC 4.0 license, https://creativecommons.org/licenses/by-nc/4.0/

Here, we report a similar case from Lepidoptera, family Geometridae. *Drepanogynis insciata* (Felder & Rogenhofer, 1875) is a distinctive species with greenish-whitish wings, wine-red margins, and a relatively large wingspan of 27 mm (Figs 1, 2). This species was originally described from South Africa in 1875 based on a single male specimen. Although the holotype lacks a collection date (Fig. 1A), there is good reason to believe it was collected in 1857 during the Austrian frigate’s (*Fregatte Novara*) global expedition. The explorers made their long voyage during 1857–1859 and visited South Africa from 2 to 26 October 1857 (Wüllerstorf-Urbair 1861: 174–226). The holotype was collected from Swellendam, the third oldest town in South Africa. At the time of the Austrian expedition, Swellendam was a well-known settlement frequently visited by many travelers and naturalists (Wikipedia 2025a).

A second historical male specimen exists in the Natural History Museum, London, although it lacks collection data (Fig. 1B). This specimen was originally part of the collection of the French entomologist Jean-Baptiste Boisduval (1799–1879), later acquired by French Charles Oberthür (1845–1924), and ultimately donated to the Natural History Museum, London in 1927 (Wikipedia 2024, 2025b). Therefore, based on indirect evidence, the second specimen must have been collected before Boisduval’s death in 1879.

Against expectations, 12 individuals of *D. insciata*—thought to be lost to science—were observed and photographed between 2020 and 2023 on the southern coast of Western Cape province, within about 150 km radius of the type locality. These records are available on iNaturalist, but the specimens were not physically vouchered (Fig. 2). A single male, which was attracted to an unspecified outdoor light in Gondwana Private Nature Reserve, near Mossel Bay on 28 January 2022, was vouchered for study (Fig. 1C). Based on this evidence, the species has remained undocumented in collections for at least 141 years from 1879 to 2020.

Taxonomic history of *D. insciata* has been complex. It was originally combined in *Axia* (?) (Felder & Rogenhofer 1875), it was later transferred to *Axiodes* Warren, 1894, as its type species (Warren 1894), then to *Drepanogynis* by Janse (1932), then again returned to *Axiodes* by Prout (1929), followed by Scoble (1999), and finally classified as *Drepanogynis insciata* in a revision of *Drepanogynis* by Krüger (2002). In that revisionary paper, this species was classified in the *D. insciata* group containing six species. The group is characterized by greenish wings (except *D. trachyacta* (Prout, 1922)) and partly scobinate, flattened processes of juxta in the male genitalia. Krüger (2002) also discussed the difficulties of *Drepanogynis* classification, proposing a broadened genus concept of *Drepanogynis* that synonymized five genera, including *Axiodes*, under *Drepanogynis*. This concept was approximating the earlier view of Janse (1932), but which was not adopted by Prout (1938). Currently, the taxon *insciata* is classified as *Drepanogynis insciata* (Krüger 2020; Rajaei et al. 2022), but the broad genus classification hypothesis of *Drepanogynis* remains to be tested, particularly with extensive molecular and life history data Murillo-Ramos et al. (2019).

Until now, *Drepanogynis insciata* has only been illustrated by a hand-drawn picture (Felder et al. 1875) and a greyscale photograph of the adult and a schematic drawing of the male genitalia (Krüger 2002). No molecular data of this taxon have been available to date. We use this opportunity to raise awareness on this unique case and on South African biodiversity in general. We aim to clarify its identity and provide high-resolution images of the type specimen, the newly collected specimen and its diagnostic characters, describe the collecting site—located in a highly transformed region on South Africa’s southern coast—and determine its phylogenetic position based on molecular data. We also identify its closest known relative based on DNA barcoding.

## ﻿Material and methods

### ﻿Examined material

**Holotype male**: [SOUTH AFRICA, Western Cape]: Swellendam (L. Taats); Novara CXXIX/ f. 10. Axia?/ insciata/ Flr. m. ♂ m; Rothschild/ Bequest/ B.M.1939-1.; Axia/ insciata/ type Felder; Geometridae/ gen. prep./ no. 21066; BMNH(E) # 275235/ Digitally scanned; NHMUK 010891612 (coll. Natural History Museum, London, UK).

**2 males**: [no locality data, no date data] Ex. Oberthür Coll./ Brit. Mus. 1927–3.; Ex MUSAEO/ Dris BOISDUVAL; NHMUK 010891613 (coll. Natural History Museum, London, UK). SOUTH AFRICA, Western Cape,/ Mossel Bay, Gondwana Private Nature Reserve,/ main Lodge, −34.0548 21.8997/ 330 m, 28-01-2022/ leg. C. Scott; Axiodes insciata/ (Felder & Rogenhofer, 1875); Pasi Sihvonen/ DNA sample 1561; Pasi Sihvonen/ prep. number 2914; micro-CT scan #164/ Max Söderholm (coll. Hermann Staude, South Africa).

**Examined photographs**: 12 males from South Africa: Western Cape, photos available on iNaturalist as detailed in Table 1.

**Table 1.** Observation details of 12 *Drepanogynis insciata* males as available on iNaturalist, arranged by observation site. None of these were physically vouchered for study, but their external characters agree with typical *D. insciata*. Search term for the species on iNaturalist is “*Axiodes insciata*”.

**Date**	**Exact site**	**Longitude, Latitude**	**Altitude (m)**	**Observer / iNaturalist username**
11.11.2023	Haarwegskloof, Overberg district municipality	-34.3383, 20.3260	200	Petra Broddle
29.10.2021	Haarwegskloof, Overberg district municipality	-34.3383, 20.3260	200	Grant Forbes
29.10.2021	Haarwegskloof, Overberg district municipality	-34.3383, 20.3260	200	Odette Curtis
10.10.2023	Mossel Bay, Hartebeestkuil Game Reserve	-34.0714, 22.0098	130	Eon Grobler
29.12.2021	Mossel Bay, Gondwana Private Nature Reserve	-34.0548, 21.8997	330	Cameron Scott
11.1.2022	Mossel Bay, Gondwana Private Nature Reserve	-34.0548, 21.8997	330	Cameron Scott
7.9.2023	Mossel Bay, Gondwana Private Nature Reserve	-34.0548, 21.8997	330	PG Coetsee
29.12.2021	Mossel Bay, Gondwana Private Nature Reserve	-34.0548, 21.8997	330	Kevin Koen
7.9.2023	Mossel Bay, Gondwana Private Nature Reserve	-34.0548, 21.8997	330	Kevin Koen
7.9.2023	Mossel Bay, Gondwana Private Nature Reserve	-34.0548, 21.8997	330	Jolandie3
25.9.2020	Mossel Bay, Gondwana Private Nature Reserve	-34.0548, 21.8997	330	Cameron Scott
10/2022	Overberg Renosterveld, Swellendam	-34.2372, 20.3312	170	Dirrtyharry

### ﻿Morphological analyses

Adult specimens, genitalia, and abdomens were prepared and photographed following methods summarized in Sihvonen et al. (2020). Uneverted vesica and tympanal organs were photographed *in situ* during dissection to allow optimal angle for observation and illustration. Wing venation was studied using non-destructive micro-CT scanning, as described by Souza Moraes et al. (2023). Photographs were edited in Adobe Photoshop v. CS6, and figures were compiled in CorelDRAW v. 24. The map of sampling sites and photographic records were created using QGIS v. 3.6.

### ﻿Molecular analyses

DNA was extracted from a leg of the specimen collected in 2022. The procedure for DNA extraction, purification, amplification, cleaning, and sequencing of both mitochondrial (COI) and protein-coding nuclear gene regions (Wingless, RpS5, Ca-ATPase, Nex9, and EF-1alpha) (Table 2) followed the protocols described by Sihvonen et al. (2020) and Lee et al. (2024). All molecular work was conducted at the DNA laboratory of the Finnish Museum of Natural History. PCR products were enzymatically cleaned and sequenced in the Institute for Molecular Medicine Finland (FIMM; Helsinki, Finland). Sequence alignment, cleaning, model selection, tree search strategies using maximum-likelihood (ML), node support estimation, and tree visualization also followed the aforementioned protocols.

**Table 2.** GenBank accession numbers for the new sequences used in this study.

**Voucher**	**COI**	**RpS5**	**Wgl**	**EF-1alpha**	**Ca-ATPase**	**Nex9**
PMS1561	PV937082	PV932705	PV932701	PV932703	PV932702	PV932704

Molecular data for *Drepanogynis insciata* were analysed using a maximum-likelihood approach implemented in IQ-TREE (Trifinopoulos et al. 2016), using the 1206-taxon dataset compiled by Murillo-Ramos et al. (2019). Best-fitting substitution models were selected by ModelFinder (Kalyaanamoorthy et al. 2017), with each partition assigned its own evolutionary rate. The resulting phylogenetic tree was rooted using representative species of the families Sematuridae, Epicopeiidae, Pseudobistonidae and Uraniidae. The tree was visualized and rooted in FigTree v. 1.4.3 (Rambaut 2015) and edited for presentation in Adobe Illustrator v. CS6 and CorelDRAW v. 24.

### ﻿DNA barcoding

A 658 bp region near the 5′ terminus of the COI mitochondrial gene (the standard animal DNA barcode) was analysed for the single specimen collected in 2022. Analyses were conducted using the BOLD analytical tools, including BIN (Barcode Index Number) and barcode gap analysis (Ratnasingham and Hebert 2007; Ratnasingham and Hebert 2013). Genetic divergences were calculated as the number of base differences between sequences and reported as a percentage, following the implementation in MEGA 11 (Tamura et al. 2021).

## ﻿Results

**Diagnosis.***Drepanogynis insciata* belongs to the *D. insciata* group (*sensu*Krüger 2002), containing six greenish (except blackish-brown *D. trachyacta* (Prout, 1922)), noctuid-like stout species characterized by distinct, undulating medial and postmedial lines on forewings, delimiting wide medial area. In the male genitalia the processes of juxta flattened, with partly scobinate surfaces.

### ﻿Key to species based on adults

**Table:** 

1	Forewings blackish-brown	***D. trachyacta* (see Krüger 2002)**
–	Forewings greenish-white	**2**
2	Forewing postmedial line pointing outwards on termen	***D. insciata***
–	Forewing postmedial line pointing down or inwards on termen	**3**
3	Hindwing with distinct, white-bordered postmedian line	***D. kalahariensis* (see Krüger 2002)**
–	Hindwing without distinct postmedian line	**4**
4	Forewing medial line evenly curved	***D. rhodampyx* (see Krüger 2002)**
–	Forewing medial line distinctly angled in middle	**5**
5	Cilia concolorous	***D. smaragdaria* (see Krüger 2002)**
–	Cilia chequered purple-and-green	***D. smaragdarioides* (see Krüger 2002)**

**Figure 3.**
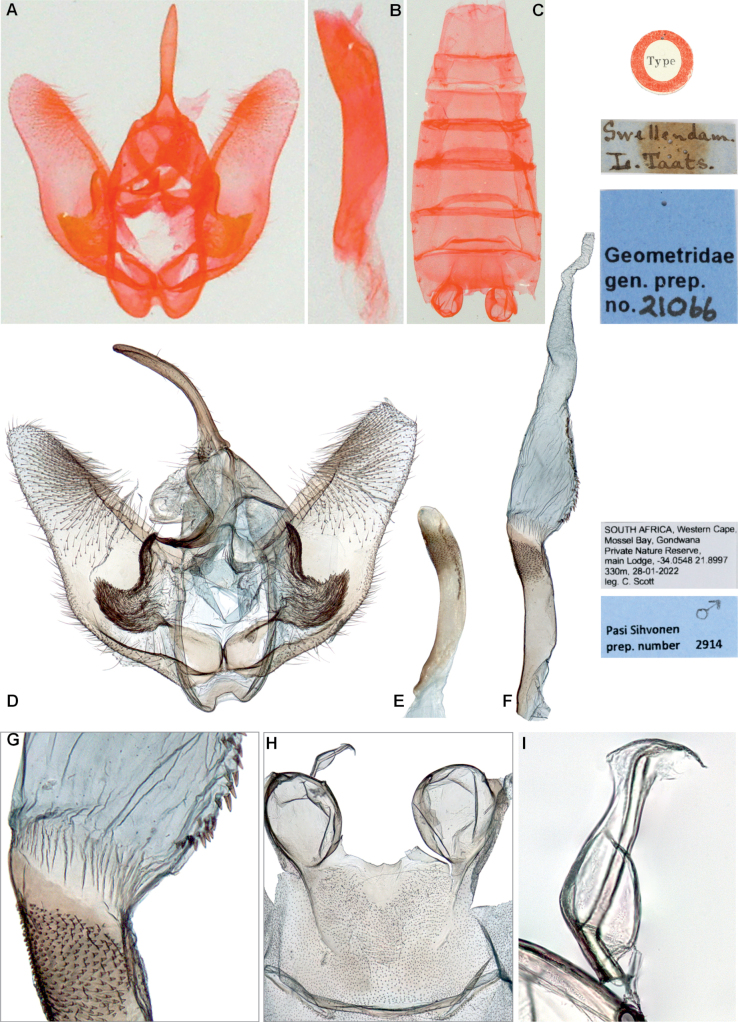
Male genitalia of *Drepanogynis insciata*. **A–C.** Holotype (coll. NHMUK); **A.** Genitalia; **B.** Aedeagus; **C.** Abdomen (dissection Geometridae gen. prep. no. 21066 by an unknown person); **D–I.** Specimen collected 28 January 2022 (coll. H. Staude); **D.** Genitalia; **E.** Aedeagus; **F.** Aedeagus with everted vesica; **G.** Magnification of the aedeagus apex with dentate sclerotisations and base of the vesica with microcornuti; **H.** Tympanal organs; **I.** Ansa (dissection Pasi Sihvonen prep. no. 2914, coll. Hermann S. Staude).

*Drepanogynis insciata* differs from all other species of *D. insciata* group (*sensu*Krüger 2002) by the distinctive shape of the forewing antemedial and postmedial lines (other species illustrated by Krüger 2002): antemedial line is concave near costa (line almost straight or angled inwards in other species), postmedial line features a pointed extrusion about halfway across the wing (extrusion round or angled in other species) and the line is angled outwards near the termen (line angled inwards or rectangular near termen in other species) (Fig. 1). Male genitalia possess unique diagnostic characters: the juxta processes are densely dentate, with a broad lateral plate and a narrow, upward-pointing extrusion (upward-pointing extrusion absent in other species). The aedeagus apex is armed with numerous minute teeth (minute teeth not illustrated for other species by Krüger 2002) (Fig. 3).

**Redescription. *External characters and abdomen***: wingspan 25–28 mm (*n* = 3). Adults rest with wings folded over body, in tent like position (Fig. 2A, B). Forewings olive green, medial area darker with red-wine-color distally, margins white. Antemedial line weakly S-shaped, with one extrusion. Postmedial line with two prominent extrusions. Hindwings basally cream, distally grey. Fringes red wine-colored, weakly chequered in both wings. Discal spots absent. Forewing costa concave. Frons, collar, and thorax concolorous with wings, abdomen pinkish dorsally. Wings below pale green, fringes and legs partly red-wine-colored. Antennae bipectinate. Hindtibia with 2 + 2 spurs. Tympanal organs medium-sized, ansa wider near base, apex with curved extension. Abdominal sternites and tergites undifferentiated. The holotype is faded in color, forewings with flesh tone.

**Figure 4.**
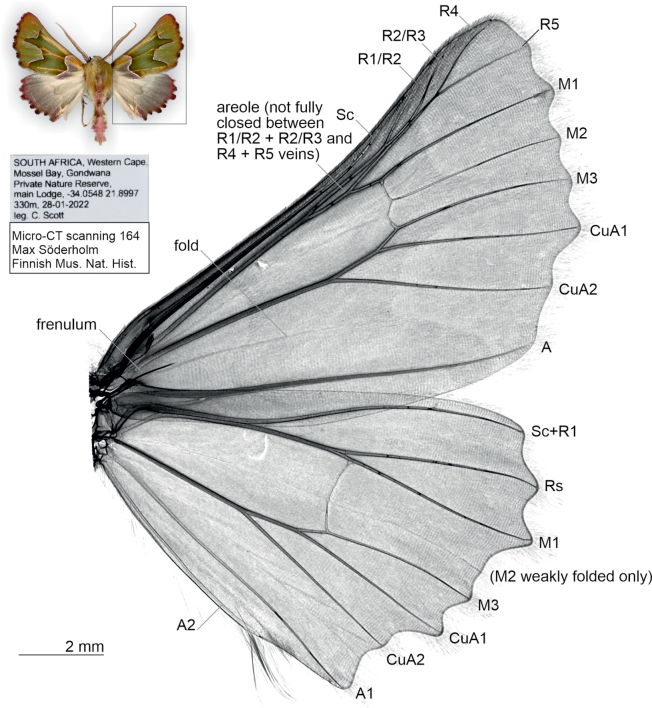
Wing venation of *Drepanogynis insciata*, specimen collected 28 January 2022 (micro-CT scan no.164 Max Söderholm, coll. Hermann S. Staude).

***Wing venation***: homology interpretation of forewing R veins is difficult. Only four veins are present as tubular, instead of common state of five. We hypothesize that one of the R1–R3 veins is fused, so that only two of these are present as tubular, and R4 + R5 are present. Forewing with four R veins: R1/R2 + R2/R3 stalked, R4 + R5 stalked, these veins running parallel, forming areole. Hindwing M2 absent, but weak fold present. Wing margins strongly crenulate (Fig. 4).

***Male genitalia***: uncus long, curved ventrally, weakly setose. Socii small, setose. Gnathos arms narrow, fused medially, forming sclerotized, sharply upcurved element. Juxta processes large, with narrow upwards pointing extrusion, lateral parts wider, upcurved, densely dentate. Valva simple, extensively setose, dorsal margin weakly sclerotized, ventral margin weakly concave. Saccus narrow, concave medially. Aedeagus weakly curved, apex covered with numerous minute teeth. Vesica opens at approximately 135° angle, basal part weakly enlarged, with row of microcornuti. Vesica apex evenly tapering (Fig. 3).

**Figure 5.**
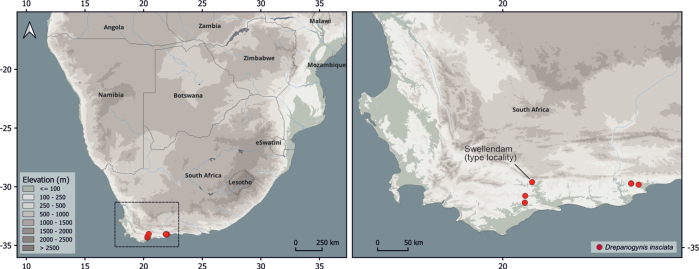
The known records of *Drepanogynis insciata* in South Africa. The species is known from five places in Western Cape only, which are within 150 km from each other. The holotype was collected from Swellendam pre-1875, likely in 1857, while all other records are between 2020 and 2023.

**Distribution and abundance.** Known from only five localities in South Africa within 150 km radius of each other: Swellendam (the type locality), Overberg Renosterveld in Swellendam, Haarwegskloof, Hartebeesstkuil Game Reserve and Gondwana Private Game Reserve, all in the Western Cape (Fig. 5). Only three male specimens are preserved in the collections: the holotype (collected before 1875) (Fig. 1A), a male collected 28.1.2022 (Fig. 1C), and a male without date, but probably collected before 1879 (Fig. 1B). Twelve male specimens were observed between 2020 and 2022, but these were not vouchered (Table 1).

**Phenology.** The species has been observed between September and January, potentially being bivoltine. Adults appear to be strictly nocturnal, and all individuals were attracted to unspecified outdoor lights.

**Biology.** Unknown.

**Figure 6.**
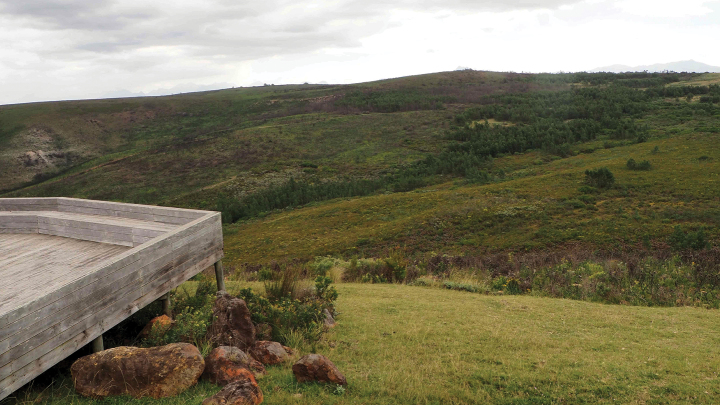
Collecting locality of *Drepanogynis insciata* in South Africa: Western Cape, Gondwana Private Nature Reserve, 330 m a.s.l., where four male specimens have been observed between 2020 and 2022. The light on the terrace was shining towards the valley. Photo taken on 29 April 2022 by Mikael Englund.

**Habitat.** Recorded from fynbos habitats at lower elevations (thus far below 330 m) on the southern slopes of the Langeberge and in the coastal plain towards the Indian Ocean from, near Swellendam and Herbertsdale. The collection locality of the specimen from the Gondwana Private Game Reserve is shown in Fig. 6.

**Figure 7.**
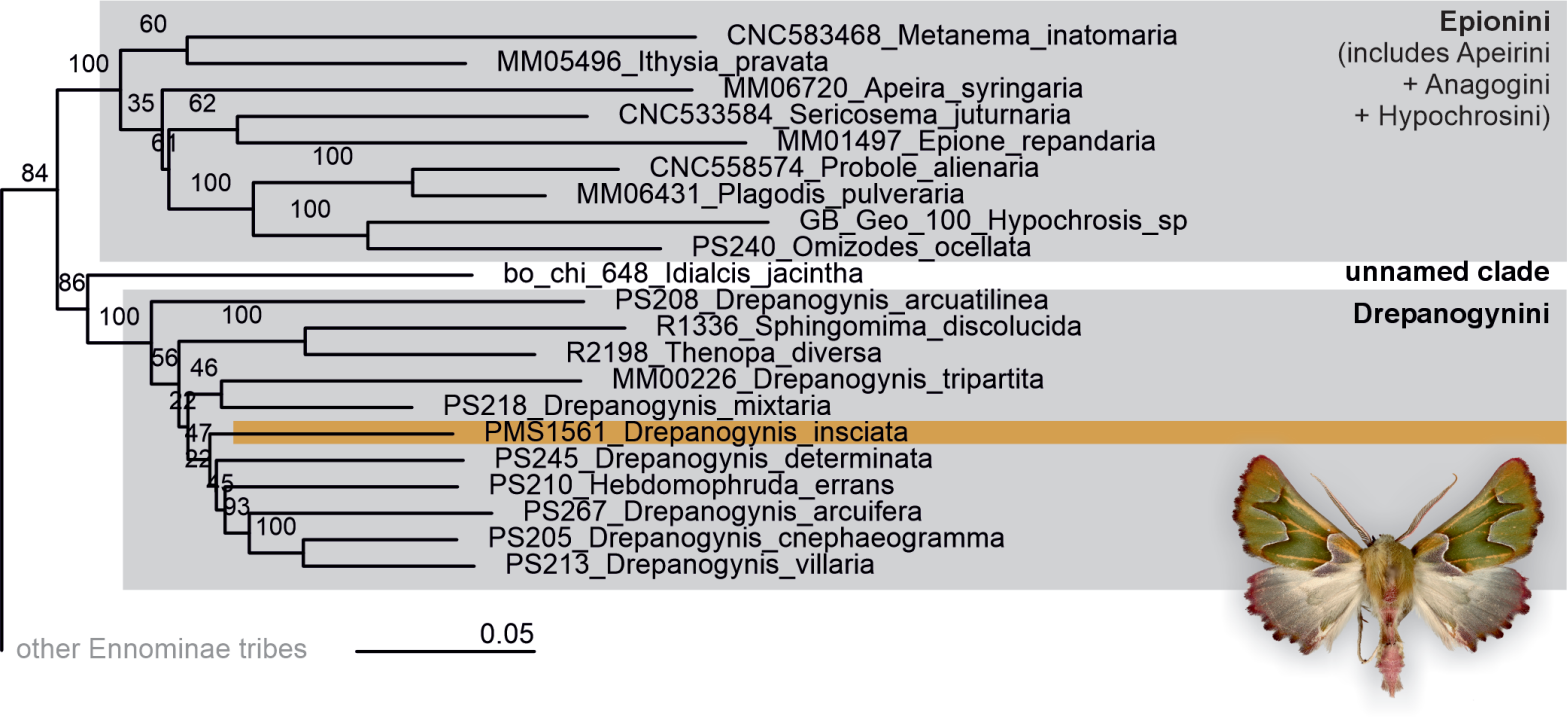
Phylogenetic position of *Drepanogynis insciata* within Ennominae: Drepanogynini in the 1206 taxa dataset of Murillo-Ramos et al. (2019) covering Geometroidea on global scale. Only relevant branches are shown, while the full tree is provided in Suppl. material 1. The tree is based on maximum-likelihood approach of up to 11 mitochondrial and nuclear genes. Numbers above branches indicate ultrafast bootstrap support. As seen on the tree, Drepanogynini genera are non-monophyletic, and those need modern revision. The classification of Epionini follows Õunap et al. (2024).

**Phylogeny and genetic data.** Based on multi-gene molecular phylogenetic analysis and morphological data, *D. insciata* is classified within Ennominae, in the tribe Drepanogynini (Fig. 7). The assignment to Drepanogynini was strongly supported in the maximum-likelihood analysis (100%), although internal relationships within the tribe were mostly poorly supported. Comparisons of the DNA barcode against data on BOLD (http://www.barcodinglife.org/) and GenBank (http://www.ncbi.nlm.nih.gov/Genbank) show the nearest genetic match to be *D. smaragdaria* Krüger, 2002 (voucher BC_ZSM_Lep_118901) with a divergence of 5.9%, and *D. bifasciata* (Dewitz, 1881) (voucher HSS-18006) with a divergence of 6.1%. However, DNA barcodes of most species of the *D. insciata* group (*sensu*Krüger 2002) are not yet available, which limits the comparative framework.

Based on the comparative morphology (see illustrations by Krüger 2002), the most similar species to *D. insciata* is *D. smaragdarioides* Krüger, 2002, both sharing the large, densely dentate juxta processes, a row of microcornuti on the vesica and chequered fringes. *Drepanogynis smaragdaria* lacks sclerotized juxta and chequered fringes.

**Conservation.** Data Deficient (IUCN Standards and Petitions Committee 2024), but probably threatened because very little of the original fynbos habitat is left on the southern coast of Western Cape, most of it has been transformed to wheatfields and grazing pastures.

**Remarks.** The broad genus concept and classification hypothesis of *Drepanogynis* proposed by Krüger (2002), which synonymizes several previously recognized genera under *Drepanogynis*, remains untested by molecular data and life history information. Recent phylogenetic work by Õunap et al. (2024) recovered *Heliomata* Grote & Robinson, 1866 as sister to Drepanogynini, but their dataset is heavily biased towards northern European taxa. In contrast, our global dataset *places Heliomata* within Nacophorini*sensu lato* lineage (Suppl. material 1), and the South American *Idialcis jacintha* Butler, 1882 was sister to Drepanogynini. *Idialcis* Warren, 1906 remains unassigned to any tribe.

## ﻿Discussion

The rediscovery of *Drepanogynis insciata* after more than 140 years raises conservation concerns. The occurrence records are sporadic, all being in a restricted area within 150 km radius with very little fynbos habitat left because it has been transformed into agricultural land (e.g. wheatfields and grazing pastures), plantation forestry and urban areas (Mucina and Rutherford 2006; Huntley and Barnard 2012). The lack of suitable habitats in a fragmented landscape may pose the biggest threat to its survival. We estimate that IUCN’s (IUCN Standards and Petitions Committee 2024) category Data Deficient (DD) best reflects the species’ current situation. To improve our understanding, we recommend monitoring the known localities and potentially including standardized light trap surveys or camera recorders as ongoing biodiversity assessments conducted more extensively in the area. Among the first steps is the identification of the caterpillar’s host plant, because this will give more precise indication about its habitat, and what kind of conservation measures best serve its survival.

South Africa harbors exceptionally rich lepidopteran diversity (e.g. Krüger 2020), yet moths remain understudied compared to for instance butterflies and vertebrates. Historical collecting and description efforts focused on charismatic or easily observable species, while nocturnal taxa have largely escaped notice (e.g. Collen et al. 2004; Essl et al. 2013; Colli et al. 2016). This bias is compounded by a shortage of taxonomic expertise (e.g. Engel et al. 2021; Hochkirch et al. 2022) and funding for moth systematics. As a result, numerous species remain undescribed, misclassified, or assumed extinct due to lack of recent records. The rediscovery of *D. insciata* is symbolic of this broader neglect and highlights the urgent need for renewed research attention and resource allocation to South Africa’s nocturnal lepidopteran fauna and invertebrate fauna more broadly.

Citizen science projects, such as iNaturalist or Caterpillar Rearing Group (Staude et al. 2020), have proven to be a valuable way to collect information on biodiversity. Their strength is in high number of participants, and data curation, making information available easily. The rediscovery of *D. insciata* is a good example of this. Without the records on iNaturalist, *D. insciata* would not have been noticed and this publication would have never been possible. While photographs have their benefits in recording the biodiversity and the distribution, our study also demonstrates that it is important to voucher physical specimens. Without the single male collected in Gondwana Private Game Reserve, we would not have the molecular data, high resolution photographs, or micro-CT scans of selected structures, and our understanding of the species would be less advanced.

Molecular data further confirm the distinctiveness of *D. insciata*, with DNA barcode showing a clear divergence from its closest relatives. However, internal relationships within the tribe Drepanogynini remain poorly resolved, likely due to insufficient taxon sampling using multi-gene data. To improve this, we are in the progress of investigating the phylogenetic relationships of *Drepanogynissensu lato* based on molecular, morphological, and ecological data and will rearrange this into multiple genera to reflect the natural relationships of the heterogeneous genus as it stands.

Additionally, the absence of barcode data for most species in the *D. insciata* group reflects a broader underrepresentation of African moths in global DNA databases. Expanding these references is essential for improving species identification and biodiversity assessments.

Ultimately, the story of *D. insciata* serves as a hopeful reminder that much remains to be discovered, and that meaningful progress is possible when field observations, citizen science, online biodiversity platforms, taxonomic expertise, and molecular tools are brought together in collaborative frameworks. We hope this rediscovery will inspire renewed efforts in documenting, protecting, and understanding the rich and enigmatic moth fauna of South Africa.

## ﻿Additional information

### Use of AI

No use of AI was reported.

### Author ORCIDs

Pasi Sihvonen https://orcid.org/0000-0003-2237-9325

Kyung Min Lee https://orcid.org/0000-0001-9055-1073

Max Söderholm https://orcid.org/0009-0003-2877-1341

Hossein Rajaei https://orcid.org/0000-0002-3940-3734

Axel Hausmann https://orcid.org/0000-0002-0358-9928

Hermann S. Staude https://orcid.org/0000-0002-2549-4372

### Data availability

All of the data that support the findings of this study are available in the main text or Supplementary Information.

## Supplementary materials

Supplementary material 1 Phylogenetic tree Data type pdf Explanation note IQ-TREE showing the full 1206 taxa dataset of Murillo-Ramos et al. (2019), based on the maximum-likelihood approach of up to 11 mitochondrial and nuclear genes, supplemented with voucher number PMS1561_Axiodes insciata (marked with pale orange). https://binary.pensoft.net/file/1482352This dataset is made available under the Open Database License (http://opendatacommons.org/licenses/odbl/1.0/). The Open Database License (ODbL) is a license agreement intended to allow users to freely share, modify, and use this Dataset while maintaining this same freedom for others, provided that the original source and author(s) are credited.Pasi Sihvonen, Kyung Min Lee, Max Söderholm, Hossein Rajaei, Axel Hausmann, Hermann S. Staude
