# Supplementary figures and images for: ﻿Drepanogynis insciata (Felder & Rogenhofer, 1875), a South African geometrid moth lost to science rediscovered after more than 140 years (Lepidoptera, Geometridae, Ennominae)
Source: Zookeys. 2025 Dec 2;1261:261–76. doi: 10.3897/zookeys.1261.171904 (PMC12690384; doi:10.3897/zookeys.1261.171904)

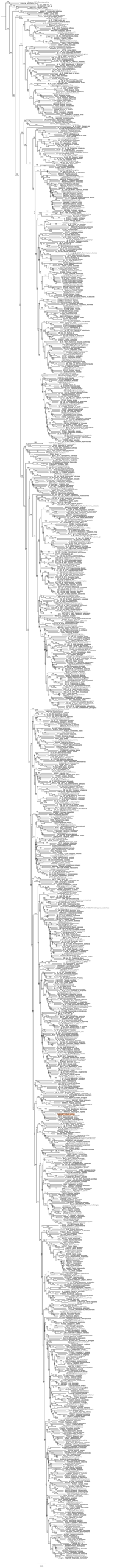

Supplement: Supplementary material 1 — Phylogenetic tree [file zookeys-1261-261_article-171904__-s001.pdf]
